# Outpatient health-seeking behavior of residents in Zhejiang and Qinghai Province, China

**DOI:** 10.1186/s12889-019-7305-0

**Published:** 2019-07-19

**Authors:** Minzhuo Huang, Hao Zhang, Yuxuan Gu, Jingming Wei, Shuyan Gu, Xuemei Zhen, Xiaoqian Hu, Xueshan Sun, Hengjin Dong

**Affiliations:** 1 Center for Health Policy Studies, Department of Social Medicine, School of Public Health, Zhejiang University School of Medicine, 866 Yuhangtang Rd, Hangzhou, 310058 People’s Republic of China; 2 Sir Run Runshaw Hospital, Zhejiang University, Hangzhou, 310016 People’s Republic of China; 3 School of Medicine, Hangzhou Normal University, Hangzhou, 311121 People’s Republic of China

**Keywords:** Health-seeking behavior, Health service utilization, Outpatient visit, Health literacy, China

## Abstract

**Background:**

The eastern and western regions of China are different in many ways such as socioeconomic characteristics and health resource distribution. This study aimed to explore the outpatient health-seeking behavior and compare the influencing factors of residents in Zhejiang and Qinghai Province, which represent the situation in eastern and western China. Thus, this research will provide evidence for health resource allocation and health reform.

**Methods:**

A cross-sectional study was conducted on a sample selected from 1600 households in Zhejiang and Qinghai province between 2016 to 2017 by the multi-stage stratified cluster random sampling method. Among the 4231 residents aged 15 years or older in the sample, 566 who reported ill-health were selected for data analysis. Two-week outpatient visits and choice of health institutions were used to measure residents’ outpatient health-seeking behavior and assessed using Chi-square tests. The binary logistic regression was adopted to demonstrate the association between explanatory variables and outpatient visits.

**Results:**

The study revealed that out of the people who reported ill-health, 58 individuals (50.97%) in Zhejiang and 106 (41.41%) in Qinghai went to health institutions to seek medical help (*p* < 0.05). The difference of residents’ choice of health institution between Zhejiang and Qinghai was not statistically significant (*p* > 0.05). Among these respondents, Self-report severity was the common and significant factor related to their outpatient visits and it had a greater impact on outpatient visits in Zhejiang (4.18, CI 2.23–7.83, *p* < 0.05). Other factors such as chronic disease, knowledge of medicine and doctors and distance to the nearest health institution were significant influencing factors in Zhejiang, while in Qinghai it was occupation.

**Conclusions:**

The outpatient health-seeking behavior and its influencing factors among residents in Zhejiang and Qinghai province were different. The findings suggest the importance of having discrepant health policies in the two provinces. It’s necessary to improve health literacy of residents in both provinces, strengthen the accessibility of health services in remote areas of Zhejiang and pay more attention to people with low socioeconomic status in Qinghai.

**Electronic supplementary material:**

The online version of this article (10.1186/s12889-019-7305-0) contains supplementary material, which is available to authorized users.

## Background

Health-seeking behavior refers to the concept, performance and actions of residents who feel unwell, have symptoms of certain diseases (even if they do not feel uncomfortable), and/or who feel the potential risk of illness and seek medical help [1–3].

In 2009, health reforms were introduced leading to progress in health services evidenced by increased investment in the health sector, expanded health insurance coverage, and better life expectancy at birth [4]. However, there are still complaints from residents about “difficulty in receiving timely medical service from a doctor and high burden of out-of-pocket expenditure [5].” It’s the right of every citizen to enjoy appropriate basic health care. Meanwhile, their health-seeking behavior is an intermediary link between the input and output of the health service system. The health service system can only bring good health output if it’s effectively used by residents [6]. Furthermore, outpatient health service utilization is usually used to express residents’ health-seeking behavior. Therefore, it can help to understand residents’ health service needs, so that the government can rationally allocate health resources and formulate sound public policies to bridge the gap between individual and different groups of health service needs and utilization [7, 8].

Existing research mainly focused on the theoretical framework of health-seeking behavior [1], the health-seeking behavior of populations in a specific area, and the health-seeking behavior of certain groups of people such as the elderly, women, chronically ill patients, and tuberculosis patients [9]. However, the eastern and western regions of China are different. Apart from the significant differences in economic development levels, the two provinces have their own unique characteristics in the geographic area, population density, cultural and health resources distribution. But few studies were found to compare residents’ health-seeking behavior in different parts of a country, especially in China. Our study selected Zhejiang and Qinghai province as areas representing eastern and western China, aimed to describe and compare health-seeking behavior of residents in the two provinces and their influencing factors in order to provide information for regional health policy making.

## Methods

### Study design and sampling

This was a cross-sectional study, which aimed to explore residents’ willingness to uptake medical services at health institutions with different administrative levels. However, this paper intended to identify residents’ outpatient health service utilization and their choice of health institutions, as well as influencing factors. Thus, this paper extracted suitable components (based on a review of the existing literature) of a larger study.

Given Zhejiang province and Qinghai province can be classified as having moderate economic development in the eastern and western regions, they were selected as sample sites. Zhejiang was an economically developed province located in southeast China, and its per capita disposable income was ¥ 38529.0 in 2016. Qinghai was an economically developing province located in the northwestern hinterland of China, and its per capita disposable income was ¥ 17301.8 in 2016 (Table 1, [10]).

**Table 1:** Social-economic characteristics of sample provinces in 2016

Province	Population (000)	Population density (/km^2^)	Per capita disposable income (¥)	Number of health personnel (/1000)
Zhejiang	5590	536.0	38529.0	7.7
Qinghai	593	8.2	17301.8	6.2
China	138271	144.3	23821.0	6.1

Source: reference [9]

Since the variance of the sample is unknown and cannot be calculated using a calculation formula, empirical sampling was used to determine the sample size. According to the usual sample size calculation method, the general sample size of regional sampling is 500–1000, so we surveyed 1000 households in Zhejiang Province. Despite the small population and low population density in Qinghai, we surveyed 600 households in Qinghai in order to have sufficient sample size for statistical calculation, resulting in a number that is larger than the sample calculated by the population size.

**Fig. 1:**
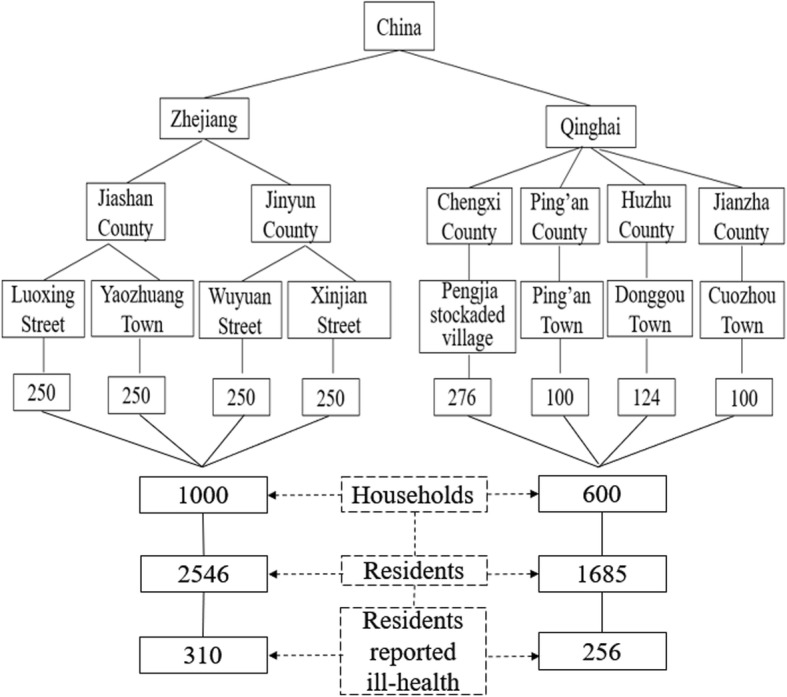
Sampling and sample size

The multi-stage stratified cluster random sampling method was adopted to choose sample sites according to the level of economic development. Firstly, Jiashan County (developed) and Jinyun County (underdeveloped) were selected in Zhejiang province, meanwhile, Chengxi County, Ping’an County (developed) and Huzhu county, Jianzha county (underdeveloped) were selected in Qinghai province. Given the small population and low population density in Qinghai, this study selected four sample counties to ensure sufficient sample size. Secondly, a town in urban areas and a street or town in rural areas in each county was randomly selected. Thirdly, based on the proportion of permanent residents, hundreds of households were randomly selected in each town or street. The sampling unit was the “household” (Fig. 1). Accordingly, Zhejiang and Qinghai provinces were investigated for 1000 and 600 households respectively. Among them, there were 2546 participants in Zhejiang Province and 1685 in Qinghai Province. Overall, 310 residents who reported ill-health in Zhejiang and 256 in Qinghai were selected for further analysis.

### Data collection

The household survey was carried out during June 2016 to August 2017. Local residents living in the region for the past 6 months and floating populations living for more than half of a year participated in face-to-face interviews conducted by investigators who used a household questionnaire. Furthermore, considering the autonomous ability of residents to seeking health services, 4231 residents aged 15 years or older were included in this study.

In order to effectively control the quality of the study, the research team contacted the health administrative departments of Zhejiang and Qinghai province in advance, and then, selected and trained high-quality investigators from local health-related departments, who were familiar with local customs. Moreover, professors and graduate students at Zhejiang University went to the sites to check the quality of the survey and verified the logic and integrity of the completed questionnaire after residents completed the questionnaires, and then provided feedback to the investigator who was responsible for the specific resident for timely correction.

### Outcome measure

Firstly, based on the reliable questionnaire of the Fifth National Health Service Survey, we designed our questionnaire (see Additional file 1). Meanwhile, we selected the following parts from the questionnaire as the research content: 1) Individual demographic characteristics and socioeconomic situation (age, gender, residence, marital status, education, income, occupation); 2) health status (self-evaluation health score, prevalence of chronic diseases in the past 6 months and the disease situation in the past 2 weeks, severity of the disease); 3) utilization of outpatient services (health institution visits and choice of health institutions); 4) satisfaction and accessibility to medical services (distance, trust in medical staff, cognition of doctor-patient relationship, knowledge of medicine and doctors). “Cognition of doctor-patient relationship” indicated that residents believed that the doctor-patient relationship was similar to friend relationships, business relationships, or other types. “Knowledge of medicine and doctors” meant that residents judged whether a doctor could solve all health problems based on their medical knowledge reserve (“yes” or “no”).

Secondly, we included residents with ill-health (i.e., “ill-health residents”) in this study. Residents who went to health institutions for treatment, to treat with drugs or other methods, went off-duty during 2 weeks prior to the survey due to the onset of an acute disease or the presence of a chronic disease were defined as having ill-health.

Thirdly, we set the two-week outpatient-visit rate and the choice of health institution as indicators to measure residents’ health-seeking behavior. It should be emphasized that in order to reduce recall bias of retrospective investigations, researchers generally use the two-week visits rather than one-month or two-month visits [11]. The two-week outpatient visit rate was used as the dependent variable. This was defined as the number of people who went to the health institution divided by the number of people with ill-health.

### Data analysis

Prior to data analysis, we coded the questionnaire and uploaded the data into a database with EpiData 3.1. Then we filled the missing data one by one according to the logic. For example, if the respondent didn’t answer the question “Do you have a chronic disease (yes or no)”, but he wrote a specific chronic disease in the following question, then we chose the option for this question as “Yes”. SPSS 21.0 was used for further analysis. Descriptive analysis, t test and Chi-square test were used to present social-demographic characteristics of sample residents, continuous variables were presented as means and standard deviation, non-continuous variables were presented as proportion. The Chi-square test was used to analyze the two-week outpatient visits of residents with ill-health and compare their choice of health institutions in Zhejiang and Qinghai. The binary logistic regression was adopted to demonstrate the association between explanatory variables and two-week outpatient visit. This study set “In the past two weeks, did you go to a health institution to rectify perceived health problems” as a dependent variable. This variable was coded as: 0 representing the resident didn’t seek or resort to a health institution when they felt ill in the past 2 weeks; 1 representing the resident went to visit a healthcare provider. A *p* value less than 0.05 was considered statistically significant.

## Results

### Social-demographic characteristics of the respondents

A total of 4231 individuals from 1600 households were surveyed, and valid individuals (15 years or above) were included in the study. The number of individuals in Zhejiang and Qinghai province were 2546 and 1685, respectively. Since this paper just explored the health-seeking behavior of residents with ill-health, so only the individuals who reported ill-health were described below. Among them, 310 residents were from Zhejiang and 256 were from Qinghai province.

Among the residents who perceived ill-health, those from Zhejiang province reported a higher health score (71.28) in general. The residents’ educational level of the two provinces was significantly different (*P* < 0.001). The number of residents who didn’t attend primary school in Qinghai province was 38.67%, which was much higher than the result of Zhejiang province (9.35%). In terms of education, over half (52.73%) of residents in Qinghai were engaged in agriculture, while Zhejiang had higher proportion of worker/administrator (36.13%), belonging to higher social-economic status. For self-reported severity, the proportion of residents in Qinghai Province (49.61%) who deemed their disease serious was nearly twice that of Zhejiang Province (26.45%). Cognition of doctor-patient relationship varied distinctly between the two provinces, more residents in Qinghai (54.3%) considered the doctor-patient relationship as friends than those in Zhejiang (30.65%). Furthermore, sociodemographic analysis indicated significant differences on residents’ knowledge of medicine and doctors, and distance to the nearest health institutions (*p* < 0.05) (Table 2).

**Table 2:** Social-demographic characteristics of residents in Zhejiang and Qinghai province (*n* = 566)

Characteristics	Zhejiang (*n* = 310)	Qinghai (*n* = 256)	Total (n = 566)	*p*
Age (Mean/SD)	61.95 (14.45)	57.50 (13.52)	59.73 (27.97)	0.116
Self-report health score (Mean/SD)	71.28 (15.19)	69.51 (17.30)	70.40 (16.25)	0.001
Gender (%)				0.115
Male	155 (50.00)	111 (43.36)	133 (46.68)	
Female	155 (50.00)	145 (56.64)	150 (53.32)	
Residence (%)				0.250
Rural	188 (60.65)	143 (55.86)	165.5 (58.25)	
Urban	122 (39.35)	113 (44.14)	117.5 (41.75)	
Marital status (%)				0.765
Never married	14 (4.52)	11 (4.30)	12.50 (4.41)	
Married	252 (81.29)	203 (79.30)	227.5 (80.29)	
Divorced or widowed	44 (14.19)	42 (16.41)	43 (15.30)	
Education (%)				< 0.001
Below primary school	29 (9.35)	99 (38.67)	64 (24.01)	
Primary school	126 (40.65)	53 (20.70)	89.5 (30.67)	
Junior middle school	89 (28.71)	66 (25.78)	77.5 (27.25)	
Senior middle school	38 (12.26)	24 (9.38)	31 (10.82)	
Junior College and above	28 (9.03)	14 (5.47)	21 (7.25)	
Income tri-quantile (%)				0.778
Low	103 (33.23)	92 (35.94)	97.5 (34.58)	
Middle	102 (32.90)	79 (30.86)	90.5 (31.88)	
High	105 (33.87)	85 (33.20)	95 (33.54)	
Occupation (%)				0.007
Student/Unemployed or semi-employed	78 (25.16)	57 (22.27)	67.5 (23.71)	
Farmer	120 (38.71)	135 (52.73)	127.5 (45.72)	
Worker/Clerk	59 (19.03)	33 (12.89)	46 (15.96)	
Administrator/Professional	53 (17.10)	31 (12.11)	42 (14.60)	
Chronic diseases (%)				0.234
Yes	241 (77.74)	188 (73.44)	214.5 (75.59)	
No	69 (22.26)	68 (26.56)	68.5 (24.41)	
Self-report severity (%)				< 0.001
Not severe	228 (73.55)	129 (50.39)	178.5 (61.97)	
Severe	82 (26.45)	127 (49.61)	104.5 (38.03)	
Trust in medical staff (%)				0.687
Very trusting	24 (7.74)	19 (7.42)	21.5 (7.58)	
Trusting	223 (71.94)	192 (75.00)	207.5 (73.47)	
General	63 (20.32)	45 (17.58)	54 (18.95)	
Cognition of doctor-patient relationship (%)				< 0.001
Friends	95 (30.65)	139 (54.30)	117 (42.47)	
Business	90 (29.03)	82 (32.03)	86 (30.53)	
Others	125 (40.32)	35 (13.67)	80 (27.00)	
Hospital or doctor can solve health problems (%)				< 0.001
No	286 (92.26)	190 (74.22)	238 (83.24)	
Yes	24 (7.74)	66 (25.78)	45 (16.76)	
Distance(kilometer)				< 0.001
< 1	172 (55.48)	192 (75.00)	182 (65.24)	
1-	59 (19.03)	33 (12.89)	46 (15.96)	
2-	27 (8.71)	20 (7.81)	23.5 (8.26)	
> 3	52 (16.77)	11 (4.30)	31.5 (10.54)	

As mentioned above, the economic level between Zhejiang and Qinghai was different, so this paper divided residents into three groups according to their household per-capita income: low, middle and high. Each group consisted of approximately 1/3 of the total residents [12]. The results showed that the per capita annual income of each group in Zhejiang was much higher than that in Qinghai Province. The high-income Group in Qinghai was similar to middle income Group in Zhejiang, which was about ¥18800. At the same time, the income share of the high-income group (72.52%) in Qinghai was higher than that in Zhejiang (66.48%), and the income share of the low-income group (7.97%) was lower than that in Zhejiang (8.33%) (Table 3).

**Table 3:** Income levels by tri-quantile

Province	Income groups	Proportion (%)	Per capita per year (¥)	Income share (%)
Zhejiang	Low (*n* = 103)	33.23	6180.53	8.33
	Middle (*n* = 102)	32.90	18868.46	25.19
	High (*n* = 105)	33.87	48359.87	66.48
Qinghai	Low (*n* = 92)	35.94	1918.29	7.97
	Middle (*n* = 79)	30.86	5468.57	19.51
	High (*n* = 85)	33.20	18892.94	72.52

### Two-week disease prevalence and outpatient health-seeking behavior

There were 310 individuals (12.18%) and 256 individuals (15.19%) in Zhejiang and Qinghai respectively that reported ill-health. Among these residents of Zhejiang, 81.94% had discomfort associated with one health problem, 14.84% presented with discomforts associated with two health problems, and 3.22% suffered from discomforts associated with three health problems. This situation was similar in Qinghai, 82.41% had only one discomfort, 12.90% mentioned two discomforts, and 4.69% suffered from three discomforts.

Considering whether these residents who went to health institutions when they encountered health problems, 158 individuals (50.97%) went to health institutions to seek medical help in Zhejiang, while this number in Qinghai was lower at106 (41.41%) (Table 4).

**Table 4:** Two-week outpatient visit of people with ill-health

Province	People with ill-health	Outpatients visit	Two-week outpatient-visit rate (%)	*χ* ^*2*^	*p*
Zhejiang	310	158	50.97	5.15	0.02
Qinghai	256	106	41.41		

As for residents’ choice of health institution, although the proportion of residents who chose primary health institutions (Village clinic/ Community health Station and health clinics in towns/Community healthcare center) in Qinghai (42.5%) was higher than that in Zhejiang (38.6%), the difference between Zhejiang and Qinghai was not statistically significant (Table 5).

**Table 5:** Residents’ choice of health institution

Province	Primary health institution	County hospital	Municipal hospitals and above	Total	*χ* ^*2*^	*p*
Zhejiang	61	68	29	158	1.41	0.49
Qinghai	45	38	23	106		

### Factors influencing residents’ outpatient visit

The results of Zhejiang province showed that chronic disease, self-reported severity, knowledge of medicine and doctors, distance to the nearest health institution were significant factors influencing outpatient visits. People with chronic disease had a lower probability of visiting health institutions than those with acute health problems. People who thought their illness was serious were four times more likely to visit a doctor. People with a higher knowledge of medicine and doctors believed that the hospital or doctor could solve all health problems. Therefore, they had higher odds of seeking health service in health institutions. There also existed evidence that people who were farther away from primary health institutions were less likely to go to health institutions when they acquired health problems.

The results in Qinghai were somewhat different from Zhejiang. Self-report severity was the only common and significant factor and it had a greater impact on two-week outpatient visits in Zhejiang. Besides, people in the profession of worker or administrator were almost three times likely to seek medical help in health institutions than students or unemployed people. This study didn’t identify a statistically significant association between health-seeking behavior and variables such as gender, residence, marital status, education, income tri-quantile and cognition of doctor-patient relationship both in Zhejiang and Qinghai (Table 6).

**Table 6:** Variables influencing residents’ two-week outpatient health visit

Explanatory variables	Odds ratio (CI 95%) (*n* = 310)	Odds ratio (CI 95%) (*n* = 256)
	Zhejiang province	Qinghai province
Gender		
Male^a^		
Female	0.88 (0.49–1.59)	1.30 (0.71–2.38)
Residence		
Rural^a^		
Urban	0.66 (0.31–1.42)	0.81 (0.38–1.73)
Marital status		
Never married^a^		
Married	0.65 (0.16–2.56)	0.90 (0.21–3.93)
Divorced or widowed	0.67 (0.13–3.42)	1.04 (0.21–5.18)
Education		
Below primary school^a^		
Primary school	1.97 (0.73–5.34)	0.88 (0.38–2.04)
Junior middle school	2.07 (0.70–6.15)	1.13 (0.49–2.61)
Senior middle school	2.67 (0.70–10.27)	1.38 (0.40–4.77)
Junior College and above	2.99 (0.62–14.37)	1.18 (0.23–6.05)
Income tri-quantile		
Low^a^		
Middle	0.41 (0.20–0.83)	1.31 (0.62–2.82)
High	0.59 (0.27–1.32)	1.31 (0.55–3.13)
Occupation		
Student/Unemployed or semi-employed^a^		
Farmer	0.93 (0.31–2.76)	1.41 (0.42–4.77)
Worker/Clerk	1.41 (0.56–3.55)	3.63 (1.28–10.28) *
Administrator/professional	1.27 (0.63–2.55)	2.58 (1.02–6.53) *
Chronic diseases		
No^a^		
Yes	0.28 (0.14–0.55) **	0.75 (0.39–1.44)
Self-report severity		
Not severe^a^		
Severe	4.18 (2.23–7.83) **	1.91 (1.06–3.44) *
Trust in medical staff		
General ^a^		
Trusting	0.86 (0.26–2.87)	1.37 (0.39–4.83)
Very trusting	0.71 (0.36–1.41)	0.69 (0.31–1.55)
Cognition of doctor-patient relationship		
Friends^a^		
Business	0.72 (0.36–1.42)	1.28 (0.57–2.85)
Others	0.97 (0.51–1.82)	1.86 (0.77–4.50)
Hospital or doctor can solve health problems		
No^a^		
Yes	2.73 (1.01–7.38) *	0.91 (0.44–1.87)
Distance to the nearest health institution (kilometer)		
< 1^a^		
1-	0.72 (0.36–1.46)	1.12 (0.49–2.54)
2-	0.35 (0.12–0.99) *	0.54 (0.18–1.59)
> 3	0.29 (0.13–0.65) **	3.63 (0.82–15.99)

^*^p < 0.05, ^**^p < 0.001

^a^Reference category

## Discussion

The highly efficient project of Universal Health Coverage calls for accessible and economic health services to meet the necessary medical needs of people [13]. Existing studies have shown that the likelihood of health treatments is different for residents of developed and developing countries when they are ill, this phenomenon also exists in developed and underdeveloped regions of a country [14–16]. The results of this study align with this research. Sick residents in Zhejiang (developed province) are more likely to visit a doctor than those in Qinghai (underdeveloped province).

Residents’ health-seeking behavior is influenced by many complicated factors, rather than isolated behavior. It usually involves factors such as socioeconomic level, cognition of the disease, and quality of health services [1, 17, 18]. Economics and health insurance appear to have no direct impact on health-seeking behavior in this study, which indicates that the outpatient service utilization was generally fair [19]. In addition, self-judgment of the severity of the disease is the common influencing factor affecting whether the residents of the two provinces will go to health institutions when they have health problems. Meanwhile, it’s known from the odds ratio of “the severity of the disease” that Zhejiang (with higher economic level) is more affected by it than Qinghai [20, 21].

There are also other factors that affect the health service utilization in the two provinces. In Zhejiang, people who have a higher level of knowledge of medicine and doctors usually believe that the doctor can solve all health problems to a greater extent, therefore, they are more likely to go to a health institution when they are sick. Therefore, we may deem that residents’ health knowledge indirectly affect their trust in doctors and thus affect their outpatient visits. Hence, it’s urgent to improve residents’ health literacy and enhance their trust in doctors. Moreover, this study reveals the lack of access to health services in Zhejiang rather than Qinghai, which is different from previous research results that developed provinces such as Zhejiang and Shanghai have better health service capabilities and accessibility [22]. However, the reasons for this should be further explored in future research. In Qinghai province, occupation affects residents’ outpatient visits. This may be due to the fact that worker/clerk and administrator/professional tend to have more social capital than farmers and unemployed individuals [23], and have a greater likelihood of being exposed to more accurate and richer medical information, and have a higher likelihood of using health services. The difference of health institutions choice between Zhejiang and Qinghai is not statistically significant. Further, although a hierarchical medical system is carried out in China, less than 50% of residents in both Zhejiang and Qinghai seek health services in primary health institutions, which means the policy of guiding residents to first go to primary health institutions needs to be further implemented.

In summary, Zhejiang and Qinghai differ in many ways, such as economic level, residents’ occupation, education, and cognition of the doctor-patient relationship. The two-week outpatient health-seeking behavior of residents and their influencing factors in the two provinces are different as well. Thus, we suggest the following recommendations to promote the health needs of residents. It is necessary to improve the health literacy of residents in both Zhejiang and Qinghai, especially for residents with low-socioeconomic status in Qinghai, which will enhance their health knowledge and the ability to seek appropriate health services as well as increase their trust in doctors when they have health problems. In addition, the hierarchical medical system needs to be better implemented to guide residents to primary health institutions to treat common diseases. For Zhejiang, the accessibility of health services in remote areas needs to be improved. For Qinghai, people with low socioeconomic status (such as farmers) need to be focused on, providing them with more access to health information and health utilization.

There exist some limitations of this study. Self-medication can be taken into accounts in further study. In addition, further study should be conducted to explore the extensive relationships between health systems and populations instead of individuals, which may help develop informed health policies.

## Conclusions

The two-week outpatient health-seeking behavior and its influencing factors among residents in Zhejiang and Qinghai province appear to be different. Residents in Zhejiang have a higher likelihood of visiting health institutions when they are ill. Outpatient visits of residents in Zhejiang are influenced by accessibility of health services and their knowledge of medicine and doctors, while residents in Qinghai are influenced by occupation. These findings suggest the need for discrepant health policies in the two provinces. It’s necessary to strengthen the accessibility of health services in remote areas of Zhejiang, pay more attention to people with low socioeconomic status in Qinghai and improve the health literacy of residents in both provinces.

## Additional file

Additional file 1: Patient medical treatment questionnaire. This file shows the household questionnaire we used in the survey, which mainly includes individual demographic characteristics and socioeconomic situation, health status, utilization of outpatient services and accessibility to medical services. (DOCX 22 kb)

## Data Availability

The datasets used and/or analysed during the current study are available from the corresponding author on reasonable request.
