# Supplementary material for: Outpatient health-seeking behavior of residents in Zhejiang and Qinghai Province, China
Source: BMC Public Health. 2019 Jul 19;19:967. doi: 10.1186/s12889-019-7305-0 (PMC6642546; doi:10.1186/s12889-019-7305-0)
Supplement: Supplementary file 1 — Patient medical treatment questionnaire. This file shows the household questionnaire we used in the survey, which mainly includes individual demographic characteristics and socioeconomic situation, health status, utilization of outpatient services and accessibility to medical services. (DOCX 22 kb) [file 12889_2019_7305_MOESM1_ESM.docx]

**Patient medical treatment questionnaire**

**Home Address: __________County (City/District) _________Township (Street) _________Village (Resident Committee)**

**Name of the owner: __________**  **Phone** **number: _____________**

**County (city/district) administrative code□□□□□□ Township(street) code□□ Village (Resident Committee) code□□**

**Household code □□□**

**Survey Start Time: __________**

**Survey Completion Time: __________**

**Verify Date: __________**

**investigator (Signed): __________**

**Investigation instructor (Signed): __________**

**Table 1. Family general situation questionnaire**

| **Number** | **Problems and options** | **Reply** |
| --- | --- | --- |
| 1 | What is the population of your household registration? (population on the household registration) |  |
| 2 | What is the number of family members living in this survey in the past 6 months? (Household population) |  |
| 3 | What is the number of non-residents living in this survey in the past 6 months? (including relatives, friends, babysitters, etc.) |  |
| 4 | What is the total income of your home in the previous year? (urban households are disposable income, rural households are net income) |  |
| 5 | How many yuan did your family spend on consumer spending in the previous year? |  |
| 6 | How much was the expenditure on medicines, medical services and supplies in consumer spending? |  |
| 7 | Is your family classified as a local poor household? (1) Yes (2) No |  |
| 8 | Is your family classified as a low-income household? (1) Yes (2) No |  |
| 9 | If you are a poor or low-income household, what do you think is the most important cause of economic hardship:  (1) Low labor force population (2) Poor natural conditions or disasters (3) Labor capacity due to disease damage (4) Treatment of diseases  (5) Unemployed or unemployed (6) Human factors (7) Others |  |

**Table 2. Family member personal situation questionnaire**

| **The code of the member under investigation (01 is the head of the household, others are coded according to the order of investigation, and once the member code is determined, it cannot be changed)** | | **01** | **02** | **03** | **04** | **05** | **06** |
| --- | --- | --- | --- | --- | --- | --- | --- |
| **A. Basic personal situation** | | | | | | | |
| 10 | Name |  |  |  |  |  |  |
| 11 | The relationship between the member and the head of the household:   1. The head of the household (2) The spouse (3) The child   (4) The son-in-law / daughter-in-law  (5) Parents (6) Grandparents/In-laws (7) Grandparents (8) Grandchildren  (9) Brother/Sister (10) Housekeeping Service Staff (11) Others |  |  |  |  |  |  |
| 12 | Account registration place:  (1) The county/district (2) The county/district outside the province  (3) Other provinces (4) Accounts to be determined |  |  |  |  |  |  |
| 13 | The nature of the household registration: (1) Agriculture (2) Non-agricultural |  |  |  |  |  |  |
| 14 | Gender: (1) Male (2) Female |  |  |  |  |  |  |
| 15 | Ethnicity: (1) Han (2) Zhuang (3) Hui (4) Uyghur (5) Mongolian (6) Tibetan  (7) Man (8) Miao (9) She (10) Others (please fill in) |  |  |  |  |  |  |
| 16 | Date of birth: Example: 19980305 |  |  |  |  |  |  |
| 17 | Marital status:  (1) Never married (2) Married (3) Separated (4) Divorce (5) Cohabitation (6) Widowed |  |  |  |  |  |  |
| 18 | Education: (1) Never go to school (2) Elementary school (3) Junior high school   1. High school (5) Technical school (6) Secondary school (7) Junior College   (8) Bachelor degree or above |  |  |  |  |  |  |
| 19 | Employment status:  (1) Employment (including flexible employment) (2) Retirement  (3) Students in school (4) Unemployed or unemployed |  |  |  |  |  |  |
| 20 | Type of occupation (inquiries about working and retirees):   1. Officials of institutions, enterprises and institutions (2) Professional and technical personnel   (3) Business/service personnel  (4) Production personnel of agriculture, forestry, animal husbandry, fishery and water conservancy industry  (5) Production and transportation equipment operators (6) Soldiers (7) Others (please fill in) |  |  |  |  |  |  |
| **B. Body function** | | | | | | | |
| 21 | Please tell the score that best represents your health today  ├---┼—-┼—-┼—-┼—-┼—-┼—-┼—-┼—-┼—-┤  0 10 20 30 40 50 60 70 80 90 100  Worst health condition Best health condition |  |  |  |  |  |  |
| **C. Chronic diseases** | | | | | | | |
| 22 | In the past 6 months, have you had a chronic disease diagnosed by a doctor?  (1) Yes (2) No (3) Unclear |  |  |  |  |  |  |
| 23 | The first chronic disease (name of disease)  (If there are multiple chronic diseases, fill in the order of severity from high to low) |  |  |  |  |  |  |
| 24 | The second chronic disease (name of disease) |  |  |  |  |  |  |
| 25 | The third chronic disease (name of disease) |  |  |  |  |  |  |

**Table 3. Investigation of illnesses and injuries within two weeks prior to the survey**

| **Code of the residents surveyed** | | 01 | 02 | 03 | 04 | 05 | 06 |
| --- | --- | --- | --- | --- | --- | --- | --- |
| 26 | Have you experienced ill-health within the first two weeks of the survey?  (1) Yes (2) No |  |  |  |  |  |  |

The following contents ask the members who have suffered from the ill-health within two weeks before the investigation. The investigators will fill in the order from the first column. If there are 2 or more kinds of discomforts, each discomfort needs to be asked, and each discomfort needs to be filled at a row. (For example, if 01 has two discomforts, then fill in two columns of information about the discomforts of 01).

| **Code of the residents surveyed** | |  |  |  |  |  |  |  |  |
| --- | --- | --- | --- | --- | --- | --- | --- | --- | --- |
| 27 | What disease or injury did you have? (fill in the name of the discomfort) |  |  |  |  |  |  |  |  |
| 28 | When did you start the discomfort this time?  (1) Newly developed within two weeks (2) Acute disease begins two weeks ago  (3) Chronic disease lasts for up to two weeks |  |  |  |  |  |  |  |  |
| 29 | If you have a discomfort in the past two weeks, what do you think is the severity of it?  (1) Serious (2) Not serious |  |  |  |  |  |  |  |  |
| 30 | In two weeks, have you been treated for this discomfort? (1) Yes (2) No |  |  |  |  |  |  |  |  |
| 31 | How many times has you been treated for this discomfort in two weeks? |  |  |  |  |  |  |  |  |
| 32 | In the next two weeks, the first type of medical institution you are attending for the illness:  (1) clinic/village clinic (2) community health service station (3) township hospital  (4) Community Health Service Center (5) County Health Center (6) Municipal health institutions (7) Provincial and above health institutions (8) Others |  |  |  |  |  |  |  |  |

**Table 4. Accessibility questionnaire for primary health care institutions**

| **Code of the residents surveyed** | | 01 | 02 | 03 | 04 | 05 | 06 |
| --- | --- | --- | --- | --- | --- | --- | --- |
| 33 | How many kilometers are the nearest medical institution to your home:  (1) less than 1 km (2) 1- (3) 2- (4) 3- (5) 4- (6) 5 km and above |  |  |  |  |  |  |
| 34 | Which of the following relationships do you think doctors and patients most closely resemble:  (1) parents and children (2) teachers and students (3) friends (4) working partners  (5) comrades (6) superiors and lowers (7) trading services (8) other |  |  |  |  |  |  |
| 35 | Do you think hospitals or doctors can solve all health problems?  (1) Yes (2) No |  |  |  |  |  |  |
